# Employment in Personality Disorders and the Effectiveness of Individual Placement and Support: Outcomes from a Secondary Data Analysis

**DOI:** 10.1007/s10926-019-09868-9

**Published:** 2019-12-09

**Authors:** T. T. Juurlink, F. Lamers, H. J. F. van Marle, H. Michon, J. T. van Busschbach, A. T. F. Beekman, J. R. Anema

**Affiliations:** 1 Psychiatry, Amsterdam Public Health Research Institute, Amsterdam UMC, Vrije Universiteit, De Boelelaan 1117, Amsterdam, The Netherlands; 2 GGZ inGeest Specialized Mental Health Care, Oldenaller 1, 1081 HJ Amsterdam, The Netherlands; 3 Trimbos Institute, Netherlands Institute of Mental Health and Addiction, Utrecht, The Netherlands; 4 University Medical Center Groningen, University Center of Psychiatry, University of Groningen, Groningen, The Netherlands; 5 Social Medicine, Amsterdam Public Health research institute, Amsterdam UMC, Vrije Universiteit, Amsterdam, The Netherlands

**Keywords:** Personality disorder, Employment, Individual placement and support, Netherlands, Secondary analysis

## Abstract

*Purpose* Personality disorders (PDs) are associated with severe functional impairment and subsequent high societal costs, increasing the need to improve occupational functioning in PD. Individual placement and support (IPS) is an effective, evidence-based method of supported employment, which so far has been tested in various mixed patient populations with severe mental illness (SMI, including PDs). However, the effectiveness of IPS for PDs per se remains uninvestigated. *Methods* Data from the SCION trial were used, including 31 SMI patients with PDs and 115 SMI patients with other primary diagnoses (primarily psychotic disorders). First, the interaction effect of diagnosis (PD vs other SMI) and intervention (IPS vs traditional vocational rehabilitation) was studied. Second, in the IPS condition, difference between diagnostic groups in time to first job was studied. *Results* We did not find evidence of a moderating effect of PD diagnosis on the primary effect of IPS (proportion who started in regular employment) (OR = 0.592, 95% CI 0.80–4.350, p = 0.606) after 30 months. Also, PD diagnosis did not moderate the effect of time until first job in IPS. *Conclusions* From the present explorative analysis we did not find evidence for a moderating effect of PD diagnosis on the effectiveness of IPS among PD participants. This indicates that IPS could be as effective in gaining employment in participants with PD as it is in participants with other SMI. Future studies, implementing larger numbers, should confirm whether IPS is equally effective in PDs and study whether augmentations or alterations to the standard IPS model might be beneficiary for PD.

## Introduction

Personality disorders (PDs) are characterized by enduring dysfunctional patterns of cognition, affect regulation, interpersonal and self-functioning, and impulse control. These dysfunctional patterns are inflexible, pervasive across a broad range of personal and social situations and cause considerable personal distress [1]. PDs affect about 6% of the general population [2] and about 45% of psychiatric outpatients [3, 4]. PDs are associated with functional impairment and unemployment [5–7]. Symptoms of PDs tend to diminish over time and PDs are responsive to treatment, however occupational functioning tends to remain poor irrespective of clinical symptom remission and adequate treatment [8, 9]. Moreover, early unemployment and functional impairment in PDs exceed that of mood and anxiety disorders [6, 10–12]. Since all PD subtypes are associated with impaired occupational functioning, it has been advocated to specifically target employment in treatment programs for PDs [13]. Currently within the Netherlands, a small number of patients with PD receive supported employment, mostly in assertive community treatment settings (not specialized in PDs). This provides an opportunity to explore the effectiveness of supported employment programs in PDs.

Hengartner and colleagues [26] showed that all PD subtypes are at least weakly associated with a low educational level, conflicts in the workplace, dismissal or demotion and unemployment. Furthermore, PDs are typically associated with deficits in interpersonal functioning characterized by a solitary lifestyle, conflictual and distressful social relations and lack of social support [14]. In persons with PDs, difficulties in gaining and maintaining employment could be related to specific deficits in interpersonal functioning. This may require adjusted or additional strategies to a standard supported employment model.

A well-established evidence-based method of supported employment is Individual Placement and Support (IPS), which originally focused on participants with severe mental illnesses (SMI) [15]. The method centers on the principle of direct employment without preceding training. Furthermore, it focusses on participants’ preferences and the assumption that everyone with a wish to gain employment should have the opportunity to find regular paid employment [16–18]. So far, IPS has been studied in various groups, such as patients with psychotic and affective disorders, veterans and patients within forensic mental health care [19–25]. Lack of information about PDs in IPS studies may be due to under-detection of PD in this population.

In short, it remains unknown whether IPS is as effective for patients with PDs as for other patients with SMI. Therefore, the aim of this study is to explore whether PDs moderate the effectiveness of IPS. Traditionally IPS does not address the interpersonal problems hindering participants with PDs [15]. Therefore, we hypothesize that IPS is less effective in PD as compared to other SMI resulting in a lower number of participants finding competitive employment. Furthermore, since PDs are associated with conflicts in the workplace and dismissal and demotion [13], we expect that participants with PD have a longer time to gaining employment compared to participants with SMI.

## Methods

### Design

Data from the first multisite randomized controlled trial studying IPS in the Netherlands (a Study of Cost-effectiveness of IPS on Open employment in the Netherlands, [SCION]) were used to perform a secondary data analysis. The SCION study was registered in the Netherlands Trial Register (Trial ID NTR292; ISRCTN87339610) [26].

### Sample and Procedures

Participants were recruited from four regional community mental health care divisions targeted at adults with severe mental illnesses. The mental health agencies operated in different areas in the Netherlands with various degrees of urbanization. Team staff consisted of psychiatrists, psychologists, community psychiatric nurses and other personnel, such as rehabilitation workers. The majority of mental health services were provided in the community, applying assertive outreach. Participants were found eligible when meeting the following criteria (1) age ranging from 18 to 65 years; (2) explicitly wishing to gain competitive employment; and (3) willing to provide informed consent. Participants were excluded when they were: (4) having paid work at study entrance; (5) full-time hospitalized; (6) engaged in another professional vocational rehabilitation program model; and (7) participating in another study with conflicting interests. All participants approved written informed consent for the study. For rationale, objectives and methods of SCION, see Michon and colleagues [26].

Participants were allocated to two comparison services, either IPS or traditional vocational rehabilitation (TVR) as the control condition (explained below). After assessing eligibility and before the start of the baseline interview participants were informed again about study consequences and asked to sign informed consent. Randomization was performed by an independent agency using a stratified block randomization procedure, with site and employment history (paid employment in the past 5 years yes/no) as stratification factors. Randomization outcomes were sent to the research team and the local research coordinators at once. Each participant received €10 (approximately $14 US) per completed interview.

For the present analysis, diagnostic information (DSM codes) had to be available. Five participants with missing DSM codes were excluded from the analyses, resulting in a total of 146 participants. Thirty-one participants were diagnosed with a PD by clinicians of the mental health agencies involved, of which 14 received IPS and 17 TVR. Of the 31 PD participants, 21 were primarily diagnosed with a PD and 10 had a secondary PD diagnosis (of which 1 paranoid PD, 1 schizoid PD, 7 borderline PD, 3 avoidant PD, 3 dependent PD, and 16 with not otherwise specified PD). Furthermore, of the 31 PD participants, 25 had concurrent Axis I disorders (of which 12 a psychotic disorder, 3 bipolar, 2 autism spectrum, 2 borderline intellectual functioning, and 6 other Axis I disorders). One-hundred-fifteen participants had no PD but had other SMI (Axis I) diagnoses (of which 56% was diagnosed with a psychotic disorder). Participants in both conditions were comparable where primary diagnoses was concerned.

### Interventions

The intervention IPS was implemented according to protocol [27], with employment specialists as members of multidisciplinary community mental health teams. Employment specialists pro-actively assisted people in gaining jobs by offering follow-along support, focused solely on regular paid employment, spending most of the time in the community and operating in close collaboration with the other community mental health team members [27].

The control condition TVR was facilitated by the mental health agency in separate rehabilitation centers or by public services. These services offer stepwise vocational trajectories, with a stronger emphasis on lengthy assessment of individual competencies and on connecting to prevocational activities such as voluntary jobs before placement in regular paid employment. These program characteristics are in contrast with the rapid job search, short assessment and minimum of prevocational training in IPS. Also, the TVR staff did not participate in the mental health teams. In the Netherlands, regardless of type of psychiatric disorder, everyone is eligible for vocational rehabilitation (zero exclusion).

During the study all sites were monitored on IPS model fidelity three times (at 6, 24 and 42 months) by means of the Quality of Supported Employment Implementation Scale (QSEIS) [28]. Two sites showed ‘good-high’ fidelity and two sites were found to have ‘moderate’ fidelity [26, 29].

### Measures

As in previous studies on IPS, the main outcome was the proportion of participants who were competitively employed during the study follow-up, dichotomously measured as having worked in competitive employment yes or no for 1 day or more [30]. In the SCION study, all outcome measures were assessed at baseline and during a 30-month follow-up period at 6, 18 and 30 months [26]. The time-points were chosen based on previous international IPS trials [18]. Diagnostic information was gathered from practitioners that were involved in the treatment of the participant (e.g. practitioner or case worker) and derived from clinical diagnoses which were made based on DSM-IV diagnostic criteria. Competitive employment was defined as having a paid job at prevailing wage, not set aside for persons with a disability, in an integrated work setting [30]. Information was derived from interviews and employment records filled out by employment specialists every two months. The employment records contained further information on dates to first job. Also quality of life by means of the MANSA [31], self-esteem by the Rosenberg Self Esteem scale [32] and the Mental Health Inventory-5 [33] for mental health were assessed during each measurement wave. Data collection procedures were identical across the control group and the intervention group.

### Analysis

An intention to treat analysis was used. Analyses were performed using SPSS (Version 24.0, Armonk, NY: IBM Corp.). For the present analysis we divided the group in participants with a personality disorder (PD) and participants with another severe mental illness (SMI) based on DSM codes provided in the dataset [34].

First, descriptive analyses were used to reveal sociodemographic similarities and differences between groups (PD versus other SMI) using the appropriate test (chi-square test, *t*-test or Mann Whitney U Test). The number of participants in competitive employment among both groups was described cumulatively by each follow-up measure in IPS and TVR. Thus, the cumulative proportion of the percentage employed at T30 means that the percentage of the considered group has found competitive employment at any time between T0 and T30. Analyses were done for each follow-up period separately as well as combined. Second, the primary outcome analysis was repeated in the present sample using logistic regression and to test the interaction of diagnosis (PD) with intervention (IPS). Third, the primary outcome of the second question was the total number of days until obtaining competitive employment during the 30-month follow-up period, serving as the dependent variable. Cox regression was used to calculate the Hazard Ratio (HR) and 95% confidence interval. The event was defined as starting a competitively employed job for the duration of at least one day. Participants were censored when they did not start a competitive job within the 30-month follow-up. If participants were lost to follow-up before starting competitive employment or the end of the study, they were censored based on the last record. For some participants the last record date extended a 30-month time period due to a prolonged interview date. This caused the analyses to be based on time periods exceeding 915 days (the average number of days in 30 months). Effect modification was investigated by the interaction term PD diagnosis × intervention (intervention vs control). All analyses used two-tailed testing procedures with 0.05 alpha levels.

## Results

### Participants

The participants of both the PD and other SMI group were equally randomized across intervention and control condition (14 IPS/17 TVR). No significant differences in baseline characteristics between groups were observed, see Table 1.

**Table 1:** Sociodemographic and clinical characteristics and self-report measures at baseline in the PD (n = 31) and other SMI group (n = 115)

	PD (n = 31)	Other SMI (n = 115)	*p*-value
Sociodemographic characteristics			
Male (%)	64.5	76.5	0.176
Mean age (SD)	36.2 (8.7)	34.6 (10.7)	0.280
Married/registered partners (%)	3.2	10.4	0.366
Paid employment in past 5 years (%)	67.7	59.1	0.383
Worked competitively in past 5 years (%)	54.8	51.3	0.727
Mean # months worked in past 5 years (SD)	24.6 (16.2)	17.6 (16.6)	0.109
Clinical characteristics			
Ever admitted to mental health hospital (%)	71.0	76.5	0.524
Self-report measures			
Mean score MANSA (self-reported quality of life) (SD)	4.2 (1.00)	4.3 (0.8)	0.811
Mean score RSE (self-reported self-esteem) (SD)	23.1 (7.1)	21.5 (4.1)	0.675
Mean score MHI-5 (self-reported mental health) (SD)	71.5 (12.7)	76.6 (11.6)	0.058

*PD* Personality Disorder, *SMI* severe mental illness

### Employment Outcomes Between Participants with PD and Other SMI

After 30 months in IPS, 35.7% of PD participants were competitively employed compared to 47.3% of other SMI participants. In TVR, 11.8% of PD participants were competitively employed compared to 25.0% of the SMI participants (Table 2). Although PD participants—both in IPS and in TVR at each follow-up—less often gained competitive employment compared to participants with other SMI, differences were not statistically significant. Note that, based on the number of participants (n = 31, n = 115) and effect sizes found in each group (0.357 and 0.473) (Cohen’s *h* = 0.24), a power calculation revealed small power 0.22 (R pwr package). Therefore, the results of our secondary, exploratory analyses should be interpreted with caution.

**Table 2:** Cumulative employment outcomes per condition in the PD (n = 31) and other SMI (n = 115) group

Employment outcomes	PD (n = 31)		Other SMI (n = 115)		*p*-value*
	IPS (n = 14)	TVR (n = 17)	IPS (n = 55)	TVR (n = 60)	
Number of individuals in intervention-arm IPS (%)	14 (45.2)	n/a	55 (47.8)	n/a	0.816
Number of persons who found competitive employment within 6 months (%)	2 (14.3)	0 (0)	13 (23.6)	8 (13.3)	0.091
Number of persons who found competitive employment within 18 months (%)	4 (28.6)	1 (5.9)	24 (43.6)	13 (21.7)	0.534
Number of persons who found competitive employment within 30 months (%)	5 (35.7)	2 (11.8)	26 (47.3)	15 (25.0)	0.459

*PD* Personality Disorder, *SMI* severe mental illness; *IPS* Individual Placement and Support, *TVR* traditional vocational rehabilitation

*Chi-square tests comparing competitive employment outcomes in intervention arm (IPS) for PD versus other SMI group; n/a: Not applicable for participants in column

### Individual Placement and Support in Personality Disorders

As previously reported by Michon and colleagues [26], we found that IPS was significantly associated with finding employment any time during follow-up (OR 0.430, 95% CI 0.216–0.857, p = 0.017). First, to test whether being diagnosed with a PD modified this outcome we added the interaction term group (PD vs other SMI) by intervention (IPS vs TVR). This interaction term was not statistically significant (OR 0.592, 95% CI 0.080–4.350, p = 0.606).

### Time to First Job in Individual Placement and Support

**Fig. 1:**
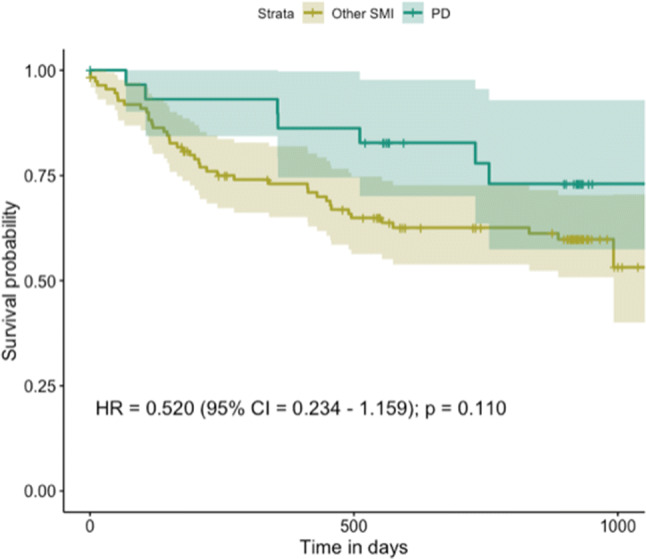
Cumulative survival of time in days to first job in IPS. PD group, group diagnosed with personality disorders; Other SMI group, group diagnosed with other severe mental illnesses; *HR* hazard ratio, *CI* confidence interval

Second, a Cox regression was performed to study the difference in time to first job between the two groups (Fig. 1). The association between having a PD diagnosis and time to first job was not significant (HR = 0.520, 95% CI 0.234–1.159, p = 0.110). Also, we did not find evidence for a moderating effect of PD on the association between IPS and time to first job (HR = 0.546, 95% CI 0.094–3.156, p = 0.499).

## Discussion

Although PDs are widespread and associated with severe impairments in occupational functioning, very little is known about the effects of standard interventions of supported employment among participants with PDs. We were able to conduct a secondary analysis testing whether PD diagnosis modifies the effect of IPS on finding a job in a RCT among participants with SMI, including a group of 31 participants with PDs. We did not find evidence of a moderating effect of PD on the primary effect of IPS on gaining employment, suggesting that IPS could be as effective in participants with PD as it is in participants with other SMI. This is important, as it would open up a much needed avenue to improve employability among people with PD.

### Interpretation of the Study Findings and Comparison with the Literature

The statistical power of the present study was too low, due to an exploratory character of the study based on post hoc exploratory analysis. Therefore, the findings should be interpreted with caution. However, contrary to our hypotheses we show that there were no differences on the primary effect of IPS and time to first job between the PD and other SMI group. This could be explained by the fact that the present study was underpowered. Yet, it may also demonstrate that it is difficult to obtain employment for persons with SMI regardless of diagnosis. However, with IPS some of the barriers to employment in SMI are alleviated, such as distance to the labor market and lack of work experience (due to illness) [35], and context related barriers such as stigma [36] and the benefits trap (the financial disincentive to return to competitive employment and thus lose social security benefits) [18, 37]. In previous studies success rates of IPS in different patient groups varied. For example, IPS in participants with SMI and justice involvement showed lower employment rates and total days of employment in IPS compared to IPS studies in SMI populations without justice involvement. Still, IPS was significantly better compared to the control condition with prevocational training and guidance [25]. Additional studies with adequate power will be needed that study whether IPS is equally effective in PD as it is in other SMI participants.

The present findings potentially indicate that participants with PD might benefit from augmentations or alterations to IPS since a lower number of participants in the PD group found competitive employment compared to the other SMI group in time to first job. Although, this difference was not significant we would like to explore potential augmentations to a standard IPS program specifically geared towards PDs. For example, it has been suggested that individuals with schizotypal PD and paranoid PD might benefit from social skills or social cognition training to improve social competence and the ability to recognize and interpret social cues in work-related situations [38]. This may also hold for other PD categories. Furthermore, effective psychotherapeutic interventions in PDs are (at least in part) geared towards challenging dysfunctional cognitions and acquiring behavioral skills to improve interpersonal and social functioning [8]. The methods used in these therapies might be partly integrated in the standard IPS program to better support PD patients and the employment specialists in assisting them. For example, employment specialists could be trained in exploring dysfunctional cognitions in stressful work-related situations with elements of cognitive behavioral therapy or aim to improve behavioral skills specifically aimed at interpersonal functioning at work.

However, the present study did not find that PD diagnosis moderated the effectiveness of IPS. The heterogeneity in PDs, such as borderline PD, antisocial PD or avoidant PD, each with its own symptoms, can make studying PDs as one group difficult. McGurk and colleagues [38] showed that patients with schizotypal and paranoid PD were most severely impaired in occupational functioning compared to other PDs due to cognitive impairment. In the present study, there was only one participant diagnosed with paranoid PD and none with schizotypal PD, conceivably due to less willingness to participate among these patients. However, other studies found all PD categories to be, at least to some extent, associated with occupational impairment [13, 39, 40]. Unfortunately, in our study, differences between PD diagnoses could not be analyzed due to the small number of participants within groups and severity was not taken into account. Furthermore, Yang and colleagues [40] suggested that not the PD diagnosis itself but the severity of the symptoms is positively related to the extent of occupational impairment. In line with most previous IPS trial samples [41–44], predominantly men were included in the present study. We did not identify previous studies examining the question as to why males are overrepresented in most IPS samples. Killackey and colleagues [45] prompted there might be cultural reasons for males to seek work more than females, and that case managers might prioritize work for males rather than for females. However, future studies should examine this question.

### Strengths and Limitations

To our knowledge this is the first exploratory study investigating the effectiveness of IPS in participants with PD as compared to other SMI. Nevertheless, there were also limitations to acknowledge. First, the power for comparing the groups studied in the present analysis was low. Specifically, the group of IPS participants with PD was small which hampers the interpretation of the findings. Second, no standardized assessment of PD was performed which affects accuracy of PD diagnoses. Third, not all PD categories were represented in this study, likely leading to under-classification and underestimation of the effects of PDs. Also, in groups with high heterogeneity, such as this PD group, it is more difficult to find moderating effects. Furthermore, from the present findings we were unable to generalize to all PDs. In addition, different PD categories have presumably different implications for occupational functioning. This is not assessed in the present study due to low numbers in the separate PD categories. Fourth, as previously argued severity of personality disorder symptoms plays a pivotal role in the degree of functional impairment [13, 46]. However in the present study severity was not assessed. Finally, it would have been informative to present other employment outcomes, such as the number of hours and days worked between groups. However, due to missing data, we had insufficient information to address these comparisons.

### Conclusions and Implications for Practice

In short, our findings suggest that there are no indications that having a PD diagnosis moderates the effect of IPS. Future studies examining the effectiveness of IPS in PD should include larger number of participants (representing all subtypes) with sufficient power to analyze the subtypes, examine multiple employment outcomes, and study whether participants with PD might benefit from specific augmentations or alterations to the standard IPS trajectory. In addition, the impact of severity of PD on outcomes could be measured, and IPS could be studied in treatment settings specifically geared towards PDs.

## References

[1] American Psychiatric Association. DSM-V. Am J Psychiatry. 2013.

[2] Huang B, Grant BF, Dawson DA, Stinson FS, Chou SP, Saha TD, Goldstein RB, Smith SM, Ruan WJ, Pickering RP (2006). Race-ethnicity and the prevalence and co-occurrence of diagnostic and statistical manual of mental disorders, fourth edition, alcohol and drug use disorders and Axis I and II disorders: United States, 2001 to 2002. Compr Psychiatry.

[3] Zimmerman M, Rothschild L, Chelminski I (2005). The prevalence of DSM-IV personality disorders in psychiatric outpatients. Am J Psychiatry.

[4] Beckwith H, Moran PF, Reilly J (2014). Personality disorder prevalence in psychiatric outpatients: a systematic literature review. Personal Ment Health.

[5] Tyrer P (2014). Personality disorders in the workplace. Occup Med (Chic Ill).

[6] Amundsen Ostby K, Czajkowski N, Knudsen GP, Ystrom E, Gjerde LC, Kendler KS, Orstavik RE, Reichborn-Kjennerud R (2014). Personality disorders are important risk factors for disability pensioning. Soc Psychiatry Psychiatr Epidemiol.

[7] Knudsen AK, Skogen JC, Harvey SB, Stewart R, Hotopf M, Moran P (2012). Personality disorders, common mental disorders and receipt of disability benefits: evidence from the British National Survey of Psychiatric Morbidity. Psychol Med.

[8] Bateman AW, Gunderson J, Mulder R (2015). Treatment of personality disorder. Lancet.

[9] Coid J, Yang MIN, Tyrer P, Roberts A, Ullrich S (2006). Prevalence and correlates of personality disorder in Great Britain. Br J Psychiatry.

[10] Skodol AE, Pagano ME, Bender DS, Shea MT, Gunderson JG, Yen S, Stout RL, Morey LC, Sanislow CA, Grilo CM, Zanarini MC, McGlashan TH (2005). Stability of functional impairment in patients with schizotypal, borderline, avoidant, or obsessive–compulsive personality disorder over two years. Psychol Med.

[11] Rymaszewska J, Jarosz-Nowak J, Kiejna A, Kallert T, Schützwohl M, Priebe S, Wright D, Nawka P, Raboch J (2007). Social disability in different mental disorders. Eur Psychiatry.

[12] Korkeila J, Oksanen T, Virtanen M, Salo P, Nabi H, Pentti J, Vahtera J, Kivimäki M (2011). Early retirement from work among employees with a diagnosis of personality disorder compared to anxiety and depressive disorders. Eur Psychiatry.

[13] Hengartner MP, Müller M, Rodgers S, Rössler W, Ajdacic-Gross V (2014). Occupational functioning and work impairment in association with personality disorder trait-scores. Soc Psychiatry Psychiatr Epidemiol.

[14] Hengartner MP, Müller M, Rodgers S, Rössler W, Ajdacic-Gross V (2013). Interpersonal functioning deficits in association with DSM-IV personality disorder dimensions. Soc Psychiatry Psychiatr Epidemiol.

[15] Drake RE, Bond GR (2011). IPS support employment: a 20-Year update. Am J Psychiatr Rehabil.

[16] Becker D, Drake R, Bond G (2011). Benchmark outcomes in supported employment. Am J Psychiatr Rehabil.

[17] Bond GR, Drake RE, Luciano A (2015). Employment and educational outcomes in early intervention programmes for early psychosis: a systematic review. Epidemiol Psychiatr Sci.

[18] Burns T, Catty J, Becker T, Drake RE, Fioritti A, Knapp M, Lauber C, Rössler W, Tomov T, van Busschbach J, White S, Wiersma D (2007). The effectiveness of supported employment for people with severe mental illness: a randomised controlled trial. Lancet.

[19] Kilian R, Lauber C, Kalkan R, Dorn W, Rössler W, Wiersma D, van Busschbach JT, Fioritti A, Tomov T, Catty J, Burns T (2012). The relationships between employment, clinical status, and psychiatric hospitalisation in patients with schizophrenia receiving either IPS or a conventional vocational rehabilitation programme. Soc Psychiatry Psychiatr Epidemiol.

[20] Luciano A, Metcalfe JD, Bond GR, Xie H, Miller AL, Riley J, O'Malley AJ, Drake RE (2016). Hospitalization risk before and after employment among adults with Schizophrenia, bipolar disorder, or major depression. Psychiatr Serv.

[21] Campbell K, Bond GR, Drake RE (2011). Who benefits from supported employment: a meta-analytic study. Schizophr Bull.

[22] Burns T, Catty J, White S, Becker T, Koletsi M, Fioritti A, Rössler W, Tomov T, van Busschbach J, Wiersma D, Lauber C (2008). The impact of supported employment and working on clinical and social functioning: results of an international study of individual placement and support. Schizophr Bull.

[23] Kukla M, McGuire AB, Salyers MP (2015). Barriers and facilitators related to work success for veterans in supported employment: a Nationwide Provider Survey. Psychiatr Serv.

[24] Talbot E, Bird Y, Russell J, Sahota K, Schneider J, Khalifa N (2018). Implementation of individual placement and support (IPS) into community forensic mental health settings: lessons learned. Br J Occup Ther.

[25] Bond GR, Kim SJ, Becker DR, Swanson SJ, Drake RE, Krzos IM, Fraser VV, O'Neill S, Frounfelker RL (2015). A controlled trial of supported employment for people with severe mental illness and justice involvement. Psychiatr Serv.

[26] Michon H, van Busschbach JT, Stant AD, van Vugt MD, van Weeghel J, Kroon H (2014). Effectiveness of individual placement and support for people with severe mental illness in The Netherlands: a 30-month randomized controlled trial. Psychiatr Rehabil J.

[27] Bond GR, Drake RE, Becker DR (2012). Generalizability of the individual placement and support (IPS) model of supported employment outside the US. World Psychiatry.

[28] Bond GR, Campbell K, Evans LJ, Gervey R, Pascaris A, Tice S, Del Bene D, Revell G (2002). A scale to measure quality of supported employment for persons with severe mental illness. J Vocat Rehabil..

[29] van Busschbach JT, Michon H, van Vugt M, Stant AD (2011). Effectiveness of individual placement and support in the Netherlands; Effectiviteit van Individuele Plaatsing en Steun in Nederland; report of a randomized controlled trial.

[30] Bond GR, Peterson AE, Becker DR, Drake RE (2012). Validation of the revised individual placement and support fidelity scale (IPS-25). Psychiatr Serv.

[31] Priebe S, Huxley P, Knight S, Evans S (1999). Application and results of the Manchester Short Assessment of Quality of Life (MANSA). Int J Soc Psychiatry.

[32] Rosenberg M (1969). Society and the adolescent self-image.

[33] Veit CT, Ware JE (1983). The structure of psychological distress and well-being in general populations. J Consult Clin Psychol.

[34.] American Psychiatric Association (2000). DSM-IV Diagnostic and statistical manual of mental disorders.

[35] Bond GR, Drake RE (2014). Making the case for IPS supported employment. Adm Policy Ment Heal Ment Heal Serv Res.

[36] Hanisch SE, Twomey CD, Szeto ACH, Birner UW, Nowak D, Sabariego C (2016). The effectiveness of interventions targeting the stigma of mental illness at the workplace: a systematic review. BMC Psychiatry.

[37] Metcalfe JD, Drake RE, Bond GR (2018). Economic, labor, and regulatory moderators of the effect of individual placement and support among people with severe mental illness: a systematic review and meta-analysis. Schizophr Bull.

[38] McGurk SR, Mueser KT, Mischel R, Adams R, Harvey PD, McClure MM, Look AE, Lueng WW, Siever LJ (2013). Vocational functioning in schizotypal and paranoid personality disorders. Psychiatry Res.

[39] Skodol AE, Gunderson JG, McGlashan TH, Dyck IR, Stout RL, Bender DS, Grilo CM, Shea MT, Zanarini MC, Morey LC, Sanislow CA, Oldham JM (2002). Functional impairment in patients with schizotypal, borderline, avoidant, or obsessive-compulsive personality disorder. Am J Psychiatry.

[40] Yang M, Coid J, Tyrer P (2010). Personality pathology recorded by severity: National survey. Br J Psychiatry.

[41] Heslin M, Howard L, Leese M, McCrone P, Rice C, Jarrett M, Spokes T, Huxley P, Thornicroft G (2011). Randomized controlled trial of supported employment in England: 2 year follow-up of the Supported Work and Needs (SWAN) study. World Psychiatry.

[42] Burns T, Yeeles K, Langford O, Montes MV, Burgess J, Anderson C (2015). A randomised controlled trial of time-limited individual placement and support: IPS-LITE trial. Br J Psychiatry.

[43] Kukla M, Bond GR, Xie H (2012). A prospective investigation of work and nonvocational outcomes in adults with severe mental illness. J Nerv Ment Dis.

[44] Reme SE, Monstad K, Fyhn T, Sveinsdottir V, Løvvik C, Lie SA, Overland S (2019). A randomized controlled multicenter trial of individual placement and support for patients with moderate-to-severe mental illness. Scand J Work Environ Heal.

[45] Killackey E, Allott K, Woodhead G, Connor S, Dragon S, Ring J (2017). Individual placement and support, supported education in young people with mental illness: an exploratory feasibility study. Early Interv Psychiatry.

[46] Tyrer P (2008). Personality disorder and public mental health. Clin Med.

